# Demonstration of > 2*π* reflection phase range in optical metasurfaces based on detuned gap-surface plasmon resonators

**DOI:** 10.1038/s41598-020-75931-8

**Published:** 2020-11-04

**Authors:** Christopher Damgaard-Carstensen, Fei Ding, Chao Meng, Sergey I. Bozhevolnyi

**Affiliations:** Centre for Nano Optics, University of Southern Denmark, Campusvej 55, DK-5230 Odense, Denmark

**Keywords:** Nanophotonics and plasmonics, Sub-wavelength optics, Two-dimensional materials

## Abstract

Plasmonic metasurfaces, representing arrays of gap-surface plasmon (GSP) resonators and consisting of arrays of metal nanobricks atop thin dielectric layers supported by thick metal films, constitute an important subclass of optical metasurfaces operating in reflection and enabling the realization of numerous, diverse and multiple, functionalities. The available phase variation range is however limited to being $<\! 2\pi$, a circumstance that complicates the metasurface design for functionalities requiring slowly varying phases over the whole range of $2\pi$, e.g., in holographic applications. The available phase range also determines the wavelength bandwidth of metasurfaces operating with linearly polarized fields due to the propagation (size-dependent) nature of the reflection phase. We suggest an approach to extend the phase range and bandwidth limitations in the GSP-based metasurfaces by incorporating a pair of detuned GSP resonators into a metasurface elementary unit cell. With detailed simulations related to those for conventional single-resonator metasurfaces and proof-of-concept experiments, we demonstrate that the detuned-resonator GSP metasurfaces designed for beam steering at ${900}\,\,\hbox {nm}$ wavelength exhibit the extended reflection phase and operation bandwidth. We believe that the considered detuned-resonator GSP metasurfaces can advantageously be exploited in applications requiring the design of arbitrary phase gradients and/or broadband operation with linearly polarized fields.

## Introduction

Wavefront-shaping using conventional optical elements relies on gradually accumulated changes of amplitude, phase or polarization during the wave propagation over many wavelengths. This generally implies using bulky components, which are not compatible with the current trend of miniaturization in photonics. Optical metasurfaces, representing nm-thin planar arrays of resonant subwavelength elements, enable complete control over spatial phase distributions of transmitted and reflected fields and thereby the realization of numerous wavefront-shaping functionalities^1–3^. Among various metasurface configurations^3,4^, plasmonic metasurfaces representing arrays of gap-surface plasmon (GSP) resonators constitute an important subclass of optical metasurfaces operating efficiently in reflection and enabling the realization of numerous, diverse and multiple, functionalities^5^. GSP-based metasurfaces consist of arrays of metal nanobricks atop thin dielectric layers deposited on optically thick metal films, with nanobrick dimensions varying in the vicinity of the GSP resonance^5–8^. The ability to control the phase, amplitude and polarization of reflected optical fields, while requiring only a single lithography step in fabrication and allowing for independent control of orthogonal linear polarizations, has attracted a great deal of attention and stimulated the recent developments of GSP-based metasurfaces^5^. The GSP metasurfaces have successfully been exploited for demonstrations of numerous functionalities, including beam steering^9–11^, planar lenses^12–15^, optical holograms^16–18^, ultrathin absorbers^19–21^, and color printing^22–24^.

The performance of optical phase-gradient metasurfaces, including the GSP-metasurfaces, is however often affected by a limitation in the available phase coverage ($<\! 2\pi$), which is related to the propagation (size-dependent) nature of the reflection phase for linearly polarized optical fields. This limitation incumbers the design of phase-gradient metasurfaces requiring slowly varying (in the metasurface plane) reflection phases of linearly polarized optical fields, e.g. in holographic applications. At the same time, the available phase range determines also the wavelength bandwidth of metasurfaces operating with linearly polarized optical fields, limiting it to a fraction of the operation wavelength (see Section 1 in Supplementary Information). The latter implies also certain constraints on the metasurface operation with ultra-short (and thus broadband) pulses of electromagnetic radiation. Taking the inspiration from previously reported theoretical studies^25,26^ and our concept of detuned electrical dipoles^27^, we propose in this work an approach to extend the phase range and bandwidth limitations in the GSP-based metasurfaces by incorporating a pair of detuned GSP resonators into a metasurface elementary unit cell. By conducting detailed numerical simulations and proof-of-concept experiments, we demonstrate that the detuned-GSP-resonator (DGSPR) metasurfaces designed for beam steering at the wavelength of ${900}\,\hbox {nm}$ exhibit the extended reflection phase and operation bandwidth. We show that, in comparison with the conventional single-resonator metasurfaces, the DGSPR metasurfaces ensure smoother phase variations and larger operation bandwidths, although at the cost of lower efficiency. We believe that the considered DGSPR metasurfaces can advantageously be exploited in applications requiring the design of arbitrary phase gradients and/or broadband operation with linearly polarized fields.

## Results

**Figure 1:**
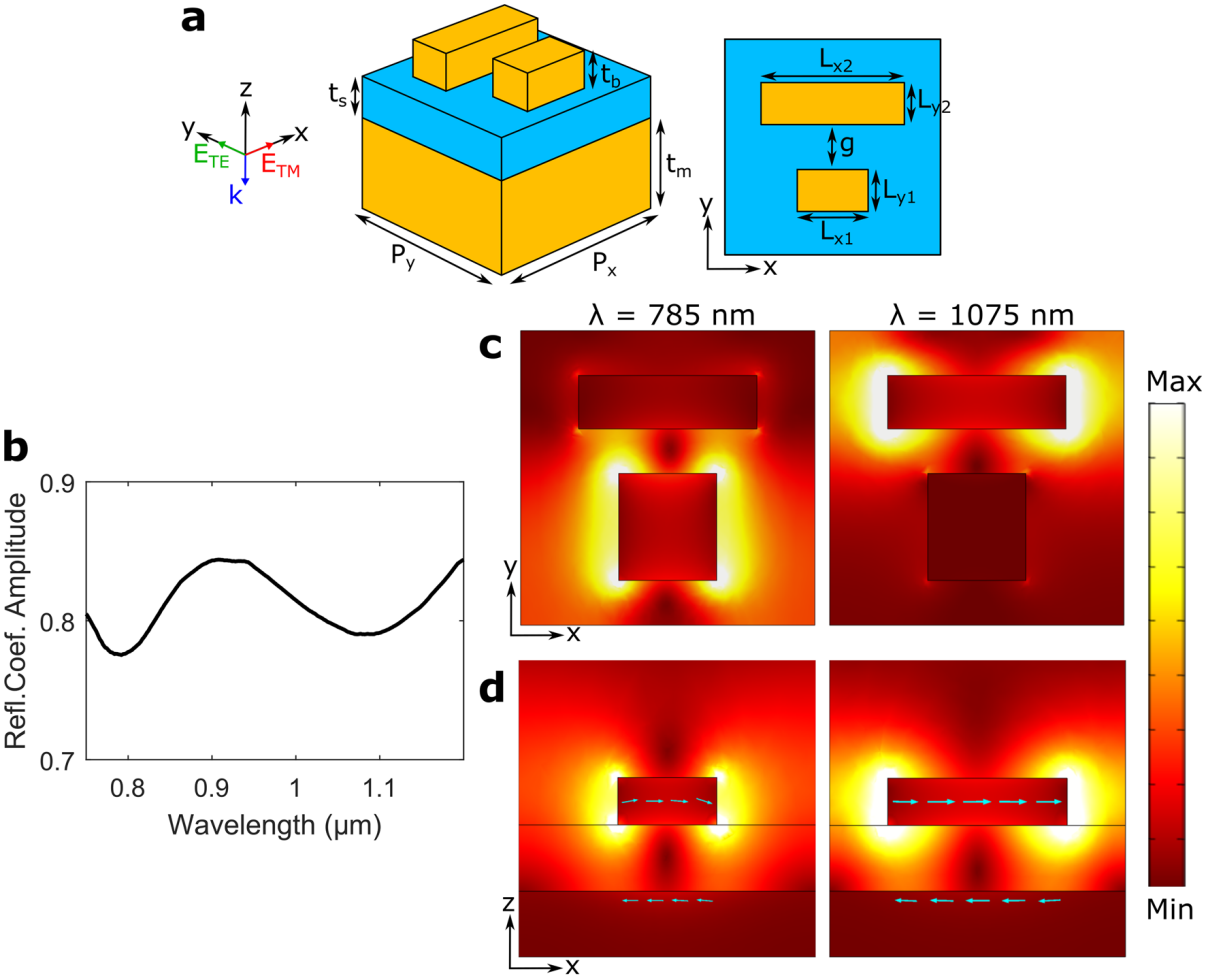
Basic unit cell sketch and investigation of detuned GSP resonators. (**a**) 3D and top-view sketches of a basic unit cell consisting of two gold nanobricks atop a thin glass spacer deposited on an optically thick gold film. (**b**) Calculated reflection coefficient amplitude as a function of wavelength for normal incident TM polarized light and a unit cell configuration of $P_x=P_y={330}\,\hbox {nm}$, $t_m = t_s = {70}\,\hbox {nm}$, $d_x={90}\,\hbox {nm}$, $t_b=g={50}\,\hbox {nm}$, $L_{x1}={110}\,\hbox {nm}$, $L_{y1}={120}\,\hbox {nm}$, $L_{y2}={60}\,\hbox {nm}$. (**c**,**d**) Color maps of the norm of the electric field in (**c**) the *xy*-plane in the center of the nanobricks and (**d**) the *xz*-plane in the center of each nanobrick for two resonant wavelengths. The arrows indicate the direction of polarization current.

**Figure 2:**
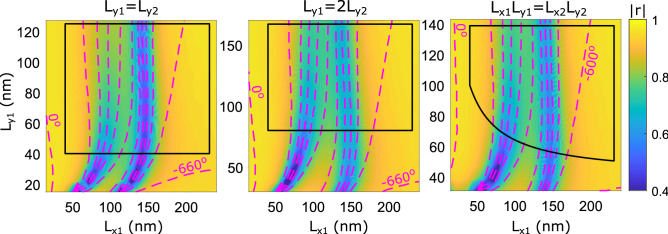
Investigation of nanobrick width relations. Calculated complex reflection coefficient, *r*, as a function of dimensions of the first nanobrick, $L_{x1}$ and $L_{y1}$, for system parameters: $P_x=P_y={330}\,\hbox {nm}$, $t_m = {70}\,\hbox {nm}$, $t_s=d_x={60}\,\hbox {nm}$, $t_b=g={40}\,\hbox {nm}$, $\lambda _0={900}\,\hbox {nm}$. The color map represents reflection coefficient amplitude and the contours represent reflected phase for normal incident TM polarized light. The separation between phase contours is ${60}^\circ$. The three color maps represent three different relations between the widths of gold nanobricks, namely equal width (left panel), the shorter nanobrick having twice the width (middle panel), and both nanobricks having equal top-surface areas (right panel).

The design of DGSPR metasurfaces involves mapping of the reflection phase and amplitude for a periodic surface array with an elementary unit cell consisting of two gold nanobricks atop a continuous dielectric (SiO_2_) spacer supported by an optically thick gold film (Fig. 1a). The nanobricks are always centered within the unit cell, with the incident light being normal to the surface and polarized along either the *x*- or *y*-direction, denoted as TM and TE polarization, respectively. In order to compose the phase-amplitude maps that are similar to those conveniently constructed for the single-resonator GSP metasurfaces^5,11,14^, only the dimensions of the short nanobrick, $L_{x1}$ and $L_{y1}$, are varied, while the dimensions of the long nanobrick depend on the short nanobrick through, $L_{x2}=L_{x1}+d_x$ (i.e., $d_x>0$ is kept constant within the considered phase-amplitude map) for the nanobrick lengths, and the relation between nanobrick widths is investigated further in Fig. 2.

The presence of DGSPRs in each elementary unit cell introduces dual GSP resonances, each corresponding to the GSP resonance of an individual nanobrick. Thus, for the considered DGSPR parameter, these resonances occur at $\sim \! {785}\,\hbox {nm}$ and $\sim \! {1085}\,\hbox {nm}$ as evidenced by two minima in the reflection coefficient amplitude (Fig. 1b). The resonant minima are relatively wide and shallow as a result of strong scattering by GSP resonators—a tunable (by adjusting the spacer thickness) and, for our purposes, desired characteristic^28^. These resonances resemble the electric dipole resonances excited by the correspondingly polarized incident field, when considering electric field spatial distributions around individual nanobricks (Fig. 1c)^29^. At the same time, the electric field distributions in the *xz*-plane (Fig. 1d) reveal the magnetic dipole nature of these resonances viewed as resonances of the nanobrick-spacer-substrate system that supports the first-order-resonant standing GSP wave characterized by the enhanced loop-circulating electric field (maximizing the magnetic field) with the antisymmetric polarization currents.

The choice of the unit cell dimensions is very important for the metasurface design, in general, and for the DGSPR metasurface design in particular, given the number of other system parameters to consider (Fig. 1a). From the viewpoint of usefulness of the phase-gradient metasurface design based on the phase-amplitude mapping conducted for periodic arrays, the intercell coupling should be minimized. The latter implies that the unit cell dimension (i.e., the array period) should satisfy: $P_{x,y}<\lambda _{\mathrm {SPP}}/2$, where $\lambda _{\mathrm {SPP}}$ is the wavelength of the corresponding surface plasmon polariton mode^30^. At the same time, the unit cell period should be large enough to allow for variation of the nanobrick widths (resulting in the phase gradient response) given aspect-ratio limitations of standard electron-beam lithography, leading to the choice of using the unit cell dimension $P_{x,y}={330}\,\hbox {nm}$ and the incident wavelength $\lambda _0={900}\,\hbox {nm}$.

The occurrence of dual GSP resonances in DGSPR metasurfaces is also reflected in the phase-amplitude maps constructed as described above. Considering the gradually increasing *x*-dimension of the short nanobrick, $L_{x1}$, the first GSP resonance occurs when the long nanobrick reaches the resonant length, followed by the second GSP resonance associated with the short nanobrick reaching the resonant length (Fig. 2). It is also immediately seen that the available phase range (i.e., the reflection phase range that can be realized by changing the nanobrick length) has indeed been increased significantly by using DGSPR metasurfaces, extending over $>\!{600}^\circ$. The initial configuration contained nanobricks of equal widths, $L_{y1}=L_{y2}$, but it turned out that the resonance of the short nanobrick was in this case highly absorbing with the phase contours being crammed close to each other (left panel in Fig. 2). We found that these deficiencies can be alleviated by increasing the short nanobrick width: a wider nanobrick featured increased reflection amplitude and spreading of phase contours, indicating that the light is reflected primarily by the nanobrick in the vicinity of GSP resonance^28^. Two approaches to increasing the short nanobrick width were tested: the short nanobrick made twice wider than the long one, $L_{y1}=2L_{y2}$, and both nanobricks to have equal top surface areas, $L_{x1}L_{y1}=L_{x2}L_{y2}$. The conducted simulations indicated that the first approach results in better matching resonant absorption and more dispersed phase contours (cf. middle and right panels in Fig. 2), so this approach will be used for further modeling. Finally, common fabrication guidelines are hereafter indicated by a black solid line marking the nanobrick dimensions corresponding to the aspect ratio (height-to-width) of one for the nanobricks and their spacing (i.e., the gap between nanobricks being equal to the nanobrick height).

**Figure 3:**
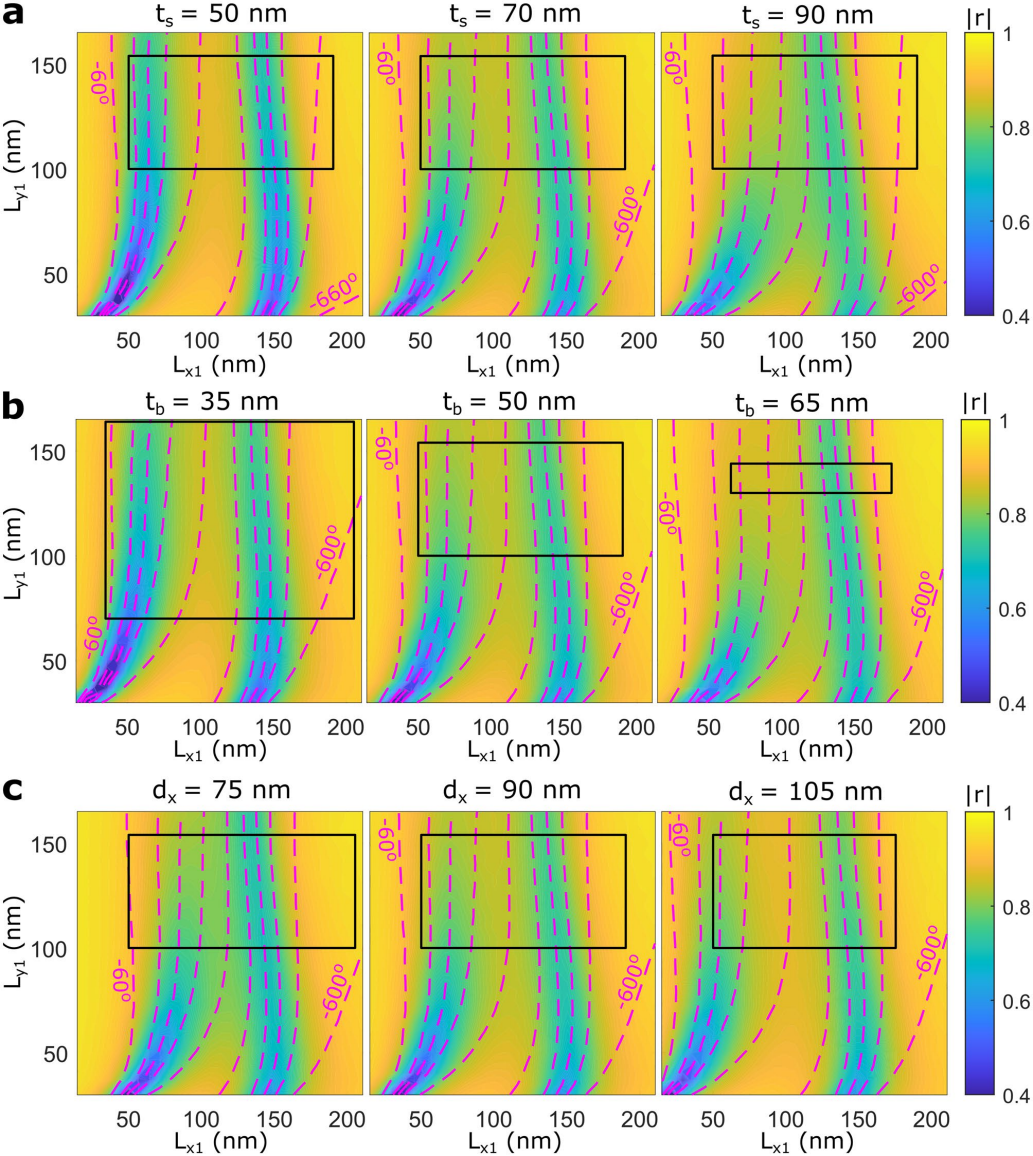
Design trend simulations for most influential system parameters. (**a**–**c**) Calculated complex reflection coefficient as a function of dimensions of the shorter nanobrick, with color maps representing reflection coefficient amplitude and contours representing reflected phase for normal incident TM polarized light. The separation between phase contours is ${60}^\circ$, and the following values are implemented for system parameters not being investigated: $P_x=P_y={330}\,\hbox {nm}$, $t_m = t_s = {70}\,\hbox {nm}$, $d_x={90}\,\hbox {nm},\; t_b=g={50}\,\hbox {nm}$, $\lambda _0={900}\,\hbox {nm}$. Investigation of the influence on the phase-amplitude map by variation of (**a**) the spacer thickness ($t_s=50,\;70,\;{90}\,\hbox {nm}$), (**b**) the nanobrick thickness ($t_b=35,\;50,\;{65}\,\hbox {nm}$), and (**c**) the difference in nanobrick length ($d_x=75,\;90,\;{105}\,\hbox {nm}$).

The phase-amplitude maps of DGSPR metasurfaces are influenced by many system parameters, of which we selected three parameters to consider highlighting the design trends (Fig. 3). The spacer thickness is known to be the crucial design parameter in GSP-based metasurfaces influencing the balance between the absorption and scattering occurring at the GSP resonance^28^. In general, smaller spacer thicknesses result in less scattering and stronger absorption at more pronounced (narrower) GSP resonances. These trends are closely related to the GSP general properties with an increase in spacer thickness leading to weaker mode confinement through a decrease in the mode effective index^31^. The trend of narrowing in the GSP resonances for smaller spacer thicknesses implies that the phase contours become crammed closer to each other, also for DGSPR metasurfaces (Fig. 3a). Increasing the spacer thickness leads consequently to spreading the phase contours, a positive tendency that however results eventually in decreasing the available phase range (Fig. 3a). Considering the influence of the nanobrick thickness, its increase enhances the GSP metasurface reflection that occurs (as noted above) primarily by the nanobrick in the vicinity of GSP resonance^28^. At the same time, increasing the nanobrick thickness reduces appreciably the area of dimensions available and thus available phase range (Fig. 3b) within given aspect-ratio-limitations mentioned previously when discussing the fabrication guidelines. These conflicting trends results in another trade-off important for the design of DGSPR metasurfaces. Finally, the difference in nanobrick lengths, $d_x$, is also a very important design parameter that controls the separation between the two GSP resonances in the phase-amplitude map by shifting the left resonance, which is the GSP resonance of the long nanobrick, to the left (right) when increased (decreased). In this case, the trade-off exist between the phase contours being too close and unevenly spaced and the available dimensions and phase range (Fig. 3c). The bottom gold film thickness and the gap between the nanobricks, $t_m$ and *g*, do not influence significantly the phase-amplitude map within the considered parameter space (Supplementary Fig. S2). We conclude the consideration of the design optimization by noting that, in all simulations shown (Fig. 3, Supplementary Fig. S2), the middle phase-amplitude map of each trend represents our choice made by balancing the reflection increase and phase contour spread. Well-separated phase contours enhance the stability against fabrication inaccuracies, while equidistant contours lead to improved broadband performance (See Section 1 in Supplementary Information). Note that this optimization approach, which is based on physical intuition and parameter sweeping, contrasts to genetic algorithm-based optimization approaches as, for example, the approach used for improving absorption and coloration in nanophotonics by means of multiple meta-atoms per unit cell^32^.

**Figure 4:**
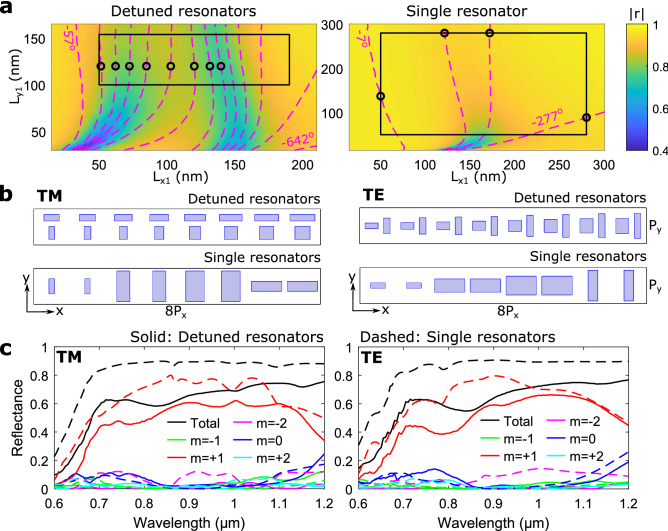
Comparison of calculated performance for DGSPR and single-resonator eight-element supercells. (**a**) Color map of calculated reflection coefficient amplitude with imposed contours representing reflected phase for DGSPR (left panel) and single-resonator (right panel) unit cells for system parameters of $P_x=P_y={330}\,\hbox {nm}$, $t_m = t_s = {70}\,\hbox {nm}$, $d_x={90}\,\hbox {nm}$, $t_b=g={50}\,\hbox {nm}$, $\lambda _0={900}\,\hbox {nm}$ and normal incident TM polarized light. For the single-resonator unit cell the $d_x$ and *g* parameters are ignored as they are meaningless, and the nanobrick is centered on the unit cell. The separation between phase contours is ${45}^\circ$ for the detuned resonators and ${90}^\circ$ for the single resonators. Circular markers represent nanobricks selected for supercell modeling. (**b**) Supercell sketches for beam steering along the positive *x*-direction for DGSPR and single-resonator configurations and for both TM and TE polarization. (**c**) Calculated diffraction efficiencies for orders $|m|\le 2$ as a function of wavelength of incident light for TM and TE polarization and for DGSPR and single-resonator supercells indicated by solid and dashed lines, respectively.

To compare the performance of DGSPR and single-GSP-resonator metasurfaces, phase-gradient metasurfaces are designed using both configurations to realize the same beam steering functionality, i.e., for anomalous (normally incident) beam reflection along the same (off-normal) direction. The designed metasurfaces may be viewed as flat blazed gratings, with the angle of first diffraction order being equal to the angle of anomalous reflection, due to the equivalence between the diffraction theory and generalized laws of reflection and refraction^33^. Continuing with the diffraction terminology, we wish to design gradient metasurfaces that reflect the incoming (at normal incidence) light into the +1st diffraction order using eight elementary unit cells for making up a supercell (resulting for the wavelength of ${900}\,\hbox {nm}$ in the diffraction angle $\theta _{r,8}\simeq {19.9}^\circ$). This may be viewed as implementing the following reflection coefficient, $r(x)=A\exp (i2\pi x/\Lambda )$, where $\Lambda$ is the supercell period of ${2640}\,\hbox {nm}$, *x* is the spatial coordinate, and *A* is the amplitude constant^11^. When designing phase gradient metasurfaces based on reflected phase ranges $<\!2\pi$, there exist several design strategies. Here, we focus on the ’equal-step phase gradient’, which demands constant phase increments, thus limiting the number of realizable individual elements. For the DGSPR and single-resonator configurations, the appropriate designs can be derived from the corresponding phase-amplitude maps (Fig. 4a), which show sequential constant-phase contours of ${45}^\circ$ and ${90}^\circ$ separation, respectively, on top of color maps of the reflection coefficient amplitude. Both maps are generated for the normally incident TM polarized light. The DGSPR phase-amplitude map indicates the available phase range of ${500}^\circ$ within the fabrication guidelines, which allows for different approaches in selecting the nanobrick dimensions (see Supplementary Fig. S3). Eight elements, indicated by black circles, are selected and sequentially arranged to form a supercell for the DGSPR metasurface (Fig. 4b). In contrast, the single-resonator map indicates the available phase range of only slightly more than ${270}^\circ$, which deems it impossible to design a supercell with eight different individual elements. It becomes necessary to duplicate each selected element (circular markers), hence the contour separation of ${90}^\circ$, to obtain a comparable eight-element supercell (Fig. 4b). The cost of duplicating elements is in using larger phase steps in the phase gradient.

Simulations of the DGSPR metasurface show that practically all reflected light is directed into the +1st diffraction order in the vicinity of the design wavelength of 900 nm, with the absolute majority of the reflected light being directed into this order within the broad wavelength range of $\sim \! {400} \,\hbox {nm}$: from $\sim \! 700$ to $\sim \!{1100} \,\hbox {nm}$ (Fig. 4c). The total reflectance of $\sim \! {65}\%$ is limited by radiation absorption (Ohmic loss) in the vicinity of the detuned GSP resonances (left panel of Fig. 4a), as the detuned resonators need to be operated near the respective resonances to realize the largest reflection phase range. In contrast, the single-resonator metasurface, which allowed to select individual elements far away from the GSP resonance (right panel of Fig. 4a), shows a total reflectance of $\sim \!{90}\%$, but the ability to suppress unintended diffraction orders is deteriorated due to the nanobrick duplication requirement. This is seen by an increase in the efficiency of −2nd diffraction order (left panel of Fig. 4c), probably because of the interference in the diffraction from two equivalent but slightly displaced sets of elements (1, 3, 5, 7) and (2, 4, 6, 8). Both sets should direct the diffracted light in the positive *x*-direction, but they might interfere constructively also in the direction of other diffraction orders. Beam steering of the TM polarization involves the light diffraction in the plane containing the polarization direction, a circumstance that may affect the diffraction performance. In order to reveal the influence of polarization on the DGSPR metasurface performance, each unit cell is rotated clockwise by ${90}^\circ$ making thereby the supercell suitable for the TE-polarized beam steering (right panel of Fig. 4b). Simulations conducted for the DGSPR and single-GSP-resonator metasurfaces operating under the TE polarized light incidence show diffraction performances very similar to those found for the TM polarized light, including poor suppression of the (unintended) −2nd diffraction order by the single-resonator metasurface (cf. left and right panels of Fig. 4c).

**Figure 5:**
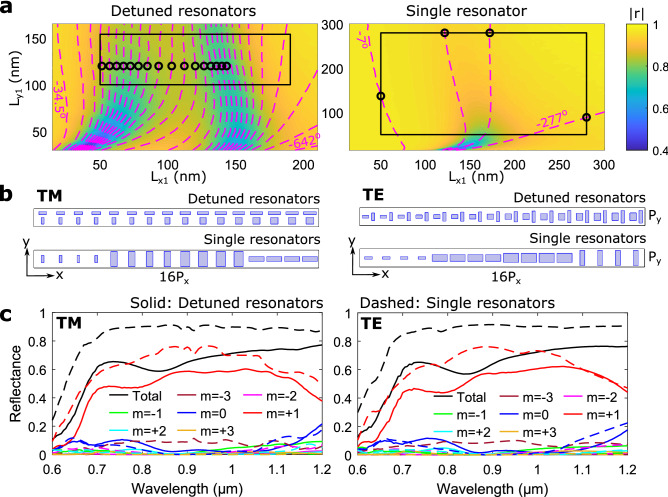
Comparison of calculated performance for DGSPR and single-resonator 16-element supercells. (**a**) Color map of calculated reflection coefficient amplitude with imposed contours representing reflected phase for DGSPR (left panel) and single-resonator (right panel) unit cells for system parameters of $P_x=P_y={330}\,\hbox {nm}$, $t_m = t_s = {70}\,\hbox {nm}, \; d_x={90}\,\hbox {nm}$, $t_b=g={50}\,\hbox {nm}$, $\lambda _0={900}\,\hbox {nm}$ and normal incident TM polarized light. For the single-resonator unit cell the $d_x$ and *g* parameters are ignored as they are meaningless, and the nanobrick is centered on the unit cell. The separation between phase contours is ${22.5}^\circ$ for the detuned resonators and ${90}^\circ$ for the single resonators. Circular markers represent nanobricks selected for supercell modeling. (**b**) Supercell sketches for beam steering along the positive *x*-direction for DGSPR and single-resonator configurations and for both TM and TE polarization. (**c**) Calculated diffraction efficiencies for orders $|m|\le 3$ as a function of wavelength of incident light for TM and TE polarization and for DGSPR and single-resonator supercells indicated by solid and dashed lines, respectively.

The advantage in using the DGSPR metasurfaces is related to their ability to generate as slowly and smoothly varying (in the metasurface plane) phase distributions as required by the design due to the inherently available reflection phase range that is extended over $2\pi$. This attractive property is investigated further by designing and comparing beam steering metasurfaces based on a 16-element supercell, resulting for the wavelength of ${900}\,\hbox {nm}$ in the first diffraction order angle $\theta _{r,16}\simeq {9.8}^\circ$. For the DGSPR metasurface, one can easily select 16 different individual elements within the available phase range that would ensure the reflection phase profile with 16 equal phase increments. For the single-resonator metasurface, one is bound to use the same four elements as before but now repeated four times each (Fig. 5a,b). Simulations of the DGSPR metasurface show diffraction performance comparable to that of the eight-element supercell metasurface considered above: close to the design wavelength of ${900}\,\hbox {nm}$, practically all reflected light is directed into the +1st diffraction order with the total reflectance being limited by highly absorbing GSP resonances (Fig. 5c). The intended +1st diffraction order efficiency extends also over ${400} \,\hbox {nm}$, being slightly more even than that of the eight-element supercell metasurface. Similarly, the single-GSP-resonator metasurface shows poor capability to suppress the unintended diffraction orders, while featuring a higher total reflectance. Notably, it is the −3rd diffraction order that is poorly suppressed for this metasurface. Supercells are designed and metasurface performances are investigated for the TE polarization as well, showing very similar performance as compared to that found for the TM polarization (cf. left and right panel of Fig. 5c).

**Figure 6:**
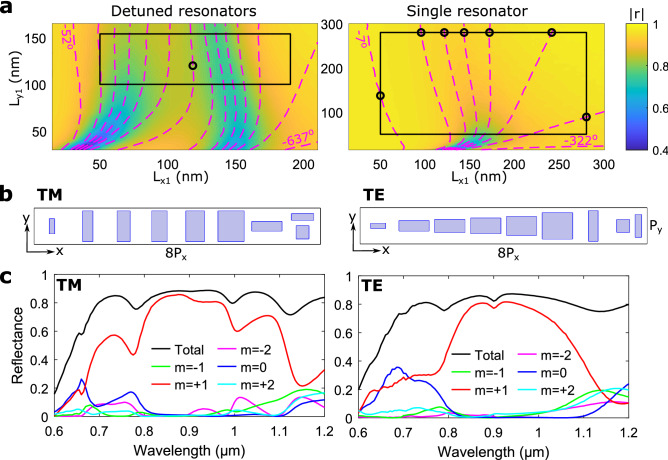
Calculated performance for a heterogeneous eight-element supercell. (**a**) Color map of calculated reflection coefficient amplitude with imposed contours representing reflected phase for DGSPR (left panel) and single-resonator (right panel) unit cells for system parameters of $P_x=P_y={330}\,\hbox {nm}$, $t_m = t_s = {70}\,\hbox {nm}$, $d_x={90}\,\hbox {nm}$, $t_b=g={50}\,\hbox {nm}$, $\lambda _0={900}\,\hbox {nm}$ and normal incident TM polarized light. For the single-resonator unit cell the $d_x$ and *g* parameters are ignored as they are meaningless, and the nanobrick is centered on the unit cell. The separation between phase contours is ${45}^\circ$ for both maps. Circular markers represent nanobricks selected for supercell modeling. (**b**) Supercell sketches for beam steering along the positive *x*-direction for heterogeneous supercells for both TM and TE polarization. (**c**) Calculated diffraction efficiencies for orders $|m|\le 2$ as a function of wavelength of incident light for TM and TE polarization.

The single-resonator GSP metasurfaces are found exhibiting superior performances in many aspects, except for the ability to suppress unintended diffraction orders, which is believed to be due to the necessity of duplicating elements because of the available reflection phase range being limited to $< \! 2\pi$. One approach to circumvent this limitation can be to combine single GSP and DGSPR unit cells in a heterogeneous metasurface, using the latter to fill the gap in the available phase range. In order to test this approach, an eight-element supercell is designed using as many individual single-GSP-resonator elements as possible and adding only those DGSPR elements that are needed to generate the out-of-range reflection phase. It turned out when considering the reflection phase range of the single-resonator unit cell that it is possible to select seven individual elements, resulting in a supercell of seven single-resonator elements and one DGSPR element (Fig. 6a,b). The metasurface performance at the design wavelength combines the attractive properties of the DGSPR and single-resonator metasurfaces, namely the suppression of unintended diffraction orders and the high total reflectance of $\sim \!{80}\%$ for both polarizations at the design wavelength of ${900} \,\hbox {nm}$ (Fig. 6c). However, the broadband performance is crippled. When introducing a DGSPR element in a supercell of single-resonator elements, the environment of several elements becomes significantly different from that existing in a periodic array of identical unit cells used in the simulations that produce the reflection phase-amplitude maps (Fig. 6a). For this reason, the performance of the considered and any other gradient metasurface might deteriorate, deviating from the expected one, due to the intercell coupling^30^. Concluding the consideration of the problematic issues arising in the design of phase-gradient GSP metasurfaces, we would like to note that there can be suggested different strategies for dealing with the limited available phase range of single-resonator GSP metasurfaces, including those that avoid duplicating nanobricks (see Section 4 in Supplementary Information).

**Figure 7:**
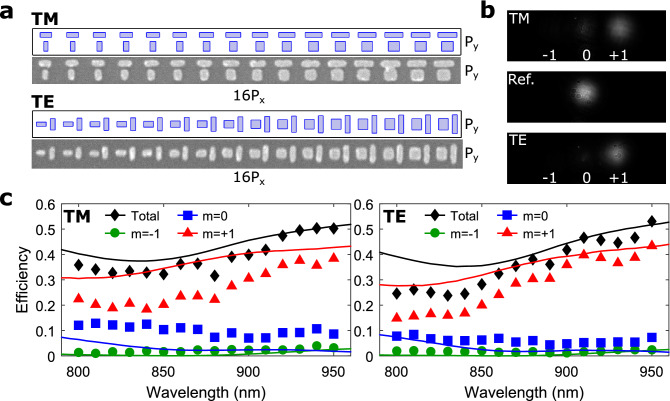
Experimental verification of the DGSPR metasurface for 16-element supercells. (**a**) Ideal and real scanning electron microscopy images of designed and fabricated supercells. (**b**) Optical images of diffraction orders for a wavelength of ${900}\,\hbox {nm}$ and for TM and TE polarization as well as a reference sample (Ref.) consisting of ${70}\,\hbox {nm}$ of SiO_2_ atop a ${70}\,\hbox {nm}$ gold substrate. (**c**) Calculated (lines) and measured (markers) diffraction efficiencies for orders $|m|\le 1$ as a function of wavelength for TM and TE polarization.

After detailed simulations and considerations of the DGSPR metasurface performance, we move on to the proof-of-concept experimental verification of the DGSPR metasurface with 16-element supercells. Two DGSPR metasurface configurations designed for operation with both TM and TE polarized light at ${900}\,\hbox {nm}$ (Fig. 5) were fabricated using the standard technological procedure based on electron-beam lithography (see “Methods” section). Scanning electron microscopy images of the fabricated supercells (Fig. 7a) indicate good correspondence between the designed and fabricated nanobrick structures, although some discrepancies/defects are seen on the fabricated DGSPR metasurfaces, including merged large nanobricks on the metasurface for TM polarization (Supplementary Fig. S5a). Visual observations of the normally incident laser beam at ${900} \,\hbox {nm}$ being reflected from unstructured sample areas and by the fabricated DGSPR metasurfaces (see “Methods” section) demonstrate that most of the reflected light is directed into the +1st (intended) diffraction order (Fig. 7b), indicating the robustness of the metasurface design against fabrication inaccuracies. Further quantifications of the metasurface performances are carried out through the determination of the 1st and 0th order diffraction efficiencies based on both simulations and experimental characterizations (Fig. 7c). For calculations of the diffraction efficiencies, the imaginary part of the complex permittivity of gold is increased by a factor of two to take into account any additional losses incurred due to various factors, including absorption in the titanium adhesion layers at gold-glass interfaces or surface scattering^11^. The results obtained for both polarizations show good correspondence between the measured and calculated diffraction efficiencies, confirming that the fabricated DGSPR metasurfaces perform in accordance with our modeling when the aforementioned additional losses are phenomenologically incorporated in the model, an approach that is widely used in practice of conventional GSP metasurfaces^5^.

## Discussion

In summary, we have suggested and considered in detail the novel design of GSP-based metasurfaces that allows one to extend the available phase range and operation bandwidth by incorporating a pair of detuned GSP resonators into a metasurface elementary unit cell. By conducting detailed numerical simulations and proof-of-concept experiments, we have demonstrated that the DGSPR metasurfaces designed for beam steering at the wavelength of ${900}\,\hbox {nm}$ exhibit the available reflection phase that is significantly wider than $2\pi$ as well as noticeably extended operation bandwidth. We have argued with numerical simulations that the very wide phase range available facilitates the design of slowly varying (in the metasurface plane) reflection phases, a feature that might be found useful for a number of applications, e.g., in holography. The extended operation bandwidth could prove beneficial in operation with ultra-short (and thus broadband) pulses of electromagnetic radiation. Thus, a simple estimation indicated that the bandwidth of $\sim \!{400}\,\hbox {nm}$ allows for undistorted operation with very short laser pulses, down to $\sim \!{7}\hbox {fs}$. Furthermore, the DGSPR metasurface performance has been compared to that of single-resonator metasurfaces designed for similar purposes, revealing different qualities of the two approaches: The single-resonator metasurface shows superior total reflectance but worse ability to suppress unwanted diffraction orders, whereas the DGSPR metasurface displays somewhat larger operation bandwidth, depending on the bandwidth criterion used, and good ability to suppress unintended diffraction orders, but at the cost of a lower total reflectance. To combine the attractive properties of both approaches, a heterogeneous metasurface was designed and numerically investigated, displaying a higher efficiency although within a narrower bandwidth. Overall, we believe that the considered detuned-resonator GSP metasurfaces can advantageously be exploited in applications requiring the design of arbitrary phase gradients and/or broadband operation with linearly polarized fields.

## Methods

### Modeling

All simulations are performed in the commercially available finite element software *COMSOL Multiphysics*, ver. 5.5. Unit and supercells are modeled to determine reflected phase-amplitude maps and diffraction efficiencies before fabrication and characterization. Application of periodic boundary conditions on the vertical sides of a cell means, it is only necessary to model a single cell. In all setups the incident wave is a plane wave traveling downward, normal to the sample surface with either TM or TE polarization. For the permittivity of gold, interpolated experimental values^34^ are used, whereas for glass, assumed to be SiO_2_, the refractive index is presumed to be purely real and take on a constant value of $n_{\text {SiO}_{2}}=1.45$. The medium above the sample is air. For the unit cell, the top and bottom boundaries of the cell are truncated by ports, which minimize any reflections. The top port, positioned an integer number of wavelengths from the nanobricks, also handles wave excitation and measures reflected light used to determine the complex reflection coefficients. All phase values represent the unwrapped phase difference between reflection from a sample with no nanobricks and a sample with nanobricks, i.e. the reflection phases in all plots are unwrapped and normalized to that produced by the reference sample with no nanobricks. For the supercell, the bottom boundary is truncated by a scattering boundary condition, and the air domain above the sample is truncated by a perfectly matched layer also to eliminate reflections. A periodic port below the perfectly matched layer handles excitation and measures the complex reflection coefficient for selected diffraction orders. Diffraction efficiencies are calculated for comparison with experimentally measured values. For this calculation, the imaginary part of the complex permittivity of gold is increased by a factor of two. This is done to take into account any additional losses related to e.g. damping in the titanium adhesion layers between gold-glass interfaces or surface scattering. The diffraction efficiencies are calculated by normalizing the amount of reflected light in a given diffraction order by the reflectance from the reference design of a ${70}\,\hbox {nm}$ layer of SiO_2_ atop a gold substrate.

### Fabrication

Fabrication of the GSP metasurfaces is done using electron beam lithography and lift-off. On a silicon substrate, ${3}\,\hbox {nm}$ of titanium is deposited using thermal evaporation followed by ${70}\,\hbox {nm}$ of gold and ${1}\,\hbox {nm}$ of titanium. Subsequently, a ${70}\,\hbox {nm}$ layer of SiO_2_ is deposited using RF-sputtering. After spin-coating of $\sim \! {100}\,\hbox {nm}$ PMMA 950K A2, the nanobricks are exposed using electron beam lithography. The exposed resist is developed and nanobricks formed by thermal evaporation of ${1}\,\hbox {nm}$ titanium and ${50}\,\hbox {nm}$ gold followed by lift-off in acetone. The three layers of titanium are for adhesion at the silicon-gold and gold-glass interfaces. All depositions are done in a Cryofox Tornado 400 system with four thermal evaporators and one RF Magnetron at average deposition rates of ${1}{\AA }\,\hbox {s}^{-1}$ for gold, ${0.1}{\AA }\,\hbox {s}^{-1}$ for Ti and ${0.45}{\AA }\,\hbox {s}^{-1}$ for SiO_2_. The metasurface size is ${26.4}\upmu \hbox {m} \times {26.4}\upmu \hbox {m}$ consisting of 5 and 80 supercells in the *x*- and *y*-direction, respectively, and it is exposed at ${30} \,\hbox {kV}$ using a JEOL JSM-6490LV electron microscope equipped with an Elphy Quantum lithography system. The fabricated structures are imaged on the same setup.

### Optical characterization

Optical characterization is performed by projecting the anomalously reflected laser beam onto a charged coupled device (CCD) to visualize the beam-steering (Supplementary Fig. S5b). Incident light is a low power, continuous-wave laser beam from a Spectra-Physics 3900 S Ti:Sapphire tunable laser at ${900}\,\hbox {nm}$, which is focused by a $20 \! \times$ objective to a spot size of $\sim \! {15}\upmu \hbox {m}$, illuminating an array of gold nanobricks while not extending beyond. The anomalously reflected light is collected by the same objective, and a beam splitter allows for separation of the incident and reflected light. A second beam splitter allows for visualization of the reflected pattern in the Fourier plane using one CCD, while simultaneously imaging the sample using a second CCD. Images of the Fourier plane are captured for the laser beam being on the array of gold nanobricks and next to it as a reference measurement for calculation of diffraction efficiency.

## Supplementary information

Supplementary Information.
