# Supplementary material for: Demonstration of > 2π reflection phase range in optical metasurfaces based on detuned gap-surface plasmon resonators
Source: Sci Rep. 2020 Nov 4;10:19031. doi: 10.1038/s41598-020-75931-8 (PMC7643121; doi:10.1038/s41598-020-75931-8)
Supplement: Supplementary file 1 — Supplementary Information. [file 41598_2020_75931_MOESM1_ESM.pdf]

Supplementary information for Demonstration of $>2\pi$ reflection phase range in optical metasurfaces based on detuned gap-surface plasmon resonators

Christopher Damgaard-Carstensen*, Fei Ding, Chao Meng, and Sergey I. Bozhevolnyi*

Centre for Nano Optics, University of Southern Denmark, Campusvej 55, DK-5230 Odense M, Denmark

*To whom correspondence should be addressed: cdc@mci.sdu.dk, seib@mci.sdu.dk

1 Wavelength dispersion of the phase response of GSP resonators

The operational bandwidth of phase-gradient metasurfaces depends on the extent of changes in their phase responses caused by tuning the operation wavelength away from the design wavelength. For the GSP-based metasurfaces, the phase response of a metasurface elementary cell is strictly related to the nanobrick length (along the polarization used) with respect to its resonant length at the design wavelength. When the nanobrick length varies near the resonant value, changes in the reflection phase are most rapid. Furthermore, the resonant nanobrick length is strictly determined by and proportional to the wavelength because the roundtrip phase is accumulated during the mode propagation in the GSP resonator [1]. When the operation wavelength is different from the design wavelength, the resonant length changes accordingly, causing the whole phase landscape to move to shorter or longer nanobrick lengths, depending on the wavelength change (Figure S1). A set of nanobricks constituting the metasurface supercell selected during the design and modeling will produce the required (stair-case) phase landscape (and the corresponding

phase gradient) at the design wavelength. A shift of the phase landscape caused by tuning the operation wavelength away from the design wavelength would inevitably change the reflection phases for all supercell nanobricks, influencing not only the available phase range but also the phase gradient in the metasurface response (Figure S1). It is seen that changes in the phase differences of neighbor nanobricks, i.e. in the phase gradient, can be rather significant even for relatively small changes in the operation wavelength (Table S1). Distortions in the phase gradient would lead to deteriorated performance and eventually determine the operation bandwidth. Phase gradient distortions can be avoided by achieving equidistant phase contours of the phase-amplitude maps, making the phase gradient insensitive to the phase landscape displacement.

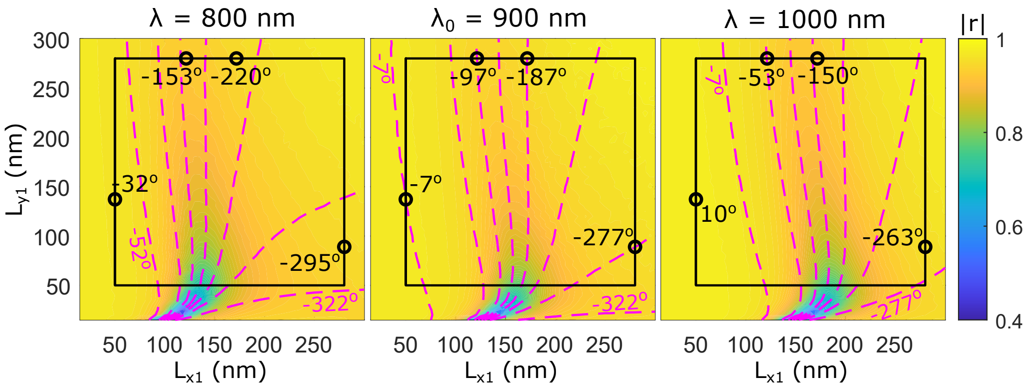

Figure S1: Color map of calculated reflection coefficient amplitude with imposed contours representing reflected phase for different wavelengths of incident light for system parameters of $P_x = P_y = 330\,\text{nm}$, $t_m = t_s = 70\,\text{nm}$, $t_b = 50\,\text{nm}$ and normal incident TM polarized light. Circular markers represent nanobricks selected for supercell modeling with corresponding reflected phase values for each selected nanobrick at the given wavelength. The separation between phase contours is 45°.

	$\Delta\varphi_{max}$	$\Delta\varphi_1$	$\Delta\varphi_2$	$\Delta\varphi_3$	$\Delta\varphi_4$
$\lambda = 800\,\text{nm}$	291°	121°	67°	75°	97°
$\lambda_0 = 900\,\text{nm}$	293°	90°	90°	90°	90°
$\lambda = 1000\,\text{nm}$	292°	63°	97°	113°	87°

Table S1: Table of the total available phase range and the phase difference between nanobricks selected for supercell modeling. $\Delta\varphi_1$ represents the phase difference between nanobrick one and two of a given panel of Figure S1, numbering from the left, $\Delta\varphi_2$ is the phase difference between nanobrick two and three, etc.

In general, a displacement of the phase landscape caused by tuning the operation wavelength changes the phase contours found within the allowed range of nanobrick dimensions

(shown by black rectangles in Figure S1), and the phase range available for other than design wavelengths would eventually become too small to ensure acceptable performance of the metasurfaces. If the phase range at the design wavelength is $>2\pi$, then the displacement of the whole phase landscape when changing the wavelength would be less critical. The additional important requirement for realizing the broadband performance is that the phase contours should be equidistant, because this displacement would then preserve intact not only the available phase range but also the phase differences in the phase response of metasurface elementary cells. The latter is needed to maintain the designed phase gradient and thereby ensure the expected metasurface performance at other than the design wavelengths.

2 Design trend simulations

As mentioned in the main text, the complex reflection coefficient depends on all the design parameters of a unit cell. The most influential parameters are covered in the main text, but here we investigate design trends for two of the less influential parameters, namely the substrate thickness, t_m, and the gap between the nanobricks, g (Figure S2). Changing the substrate thickness does not alter the phase-amplitude map, which corresponds with the requirements that the substrate should be an optically thick back-reflector. Similarly, the reflection amplitude and phase are not changed by varying the gap between the nanobricks, indicating that there is little to no coupling between the two nanobricks at the separations we are working with. As mentioned in the main text, common fabrication guidelines are indicated by the black solid line marking the nanobrick dimensions corresponding to the aspect ratio (height-to-width) of one for the nanobricks and their spacing. Note that the guidelines are broken for the $g = 35\,\text{nm}$ simulation, yet it is still relevant for investigation of the design trend.

3 Selection of nanobrick dimensions for supercell modeling

The large available reflected phase range of the detuned gap-surface plasmon resonator (DGSPR) unit cell, $\sim 500°$, offers multiple possibilities in selecting nanobrick dimensions for supercell simulations. The optimal set of design rules are determined for TM polarization and applied to all selection processes for DGSPR metasurfaces.

Investigation of the phase-amplitude maps show that selection of nanobrick dimensions

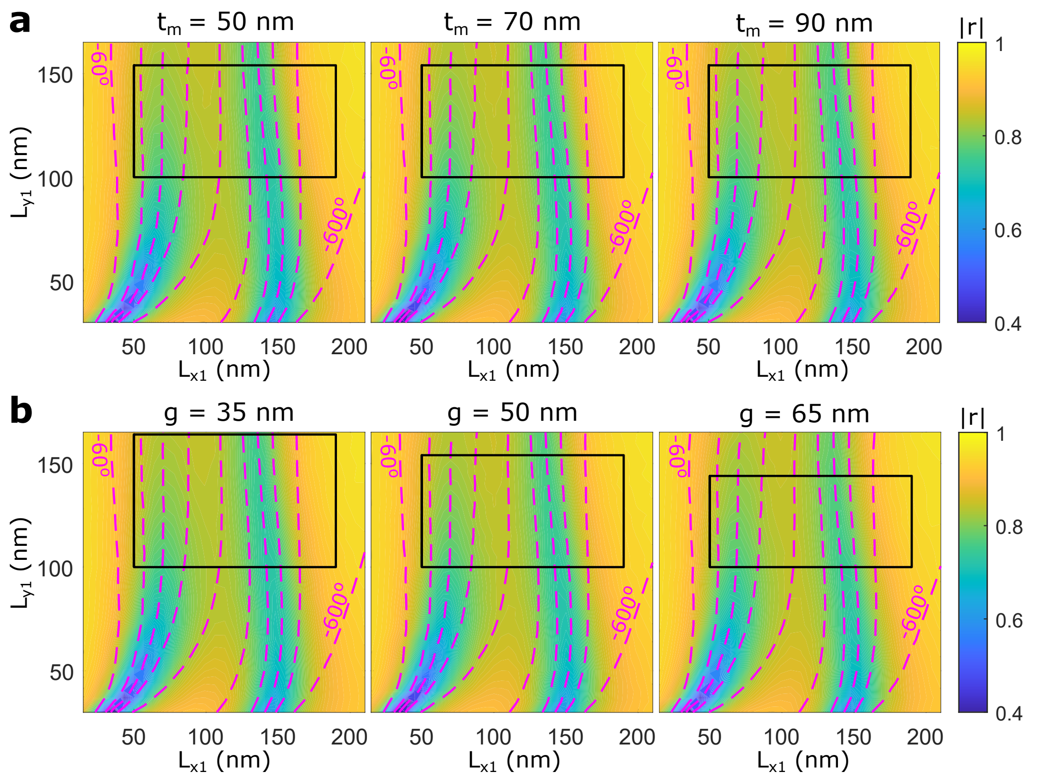

Figure S2: Design trend simulations for less influential system parameters. Calculated complex reflection coefficient as a function of dimensions of the shorter nanobrick, with color maps representing reflection amplitude and contours representing reflected phase for normal incident TM polarized light. The separation between phase contours is 60°, and the following values are implemented for system parameters not being investigated: $P_x = P_y = 330\,\text{nm}$, $t_m = t_s = 70\,\text{nm}$, $d_x = 90\,\text{nm}$, $t_b = g = 50\,\text{nm}$, $\lambda_0 = 900\,\text{nm}$. (a) Investigation of the influence on the phase-amplitude map when varying (a) the substrate thickness ($t_m = 50,\ 70,\ 90\,\text{nm}$), and (b) the gap between nanobricks ($g = 35,\ 50,\ 65\,\text{nm}$).

with the minimum separation to neighbor supercells in the y-direction (indicated by the top solid black line) leads to larger reflection coefficient amplitude (Figure S3a). This selection of 50 nm separation is compared to selecting nanobrick dimensions with twice the minimum separation, namely 100 nm, as we have found that the minimum separation leads to deteriorated performance due to unwanted coupling to neighbor supercells. The two approaches are investigated by designing eight-element supercells for a beam steering functionality at the design wavelength of 900 nm, similar to the main text. Eight elements, indicated by black circles (left and middle panels of Figure S3a), are selected and sequentially placed to form supercells for both approaches (Figure S3b). Simulations of the metasurfaces show that practically all reflected light is directed into the $+1^{\text{st}}$ diffraction order in the vicinity of the design wavelength of 900 nm. The total reflectance for the 50 nm separation approach is slightly higher corresponding to the larger widths of the nanobricks, but the ability to

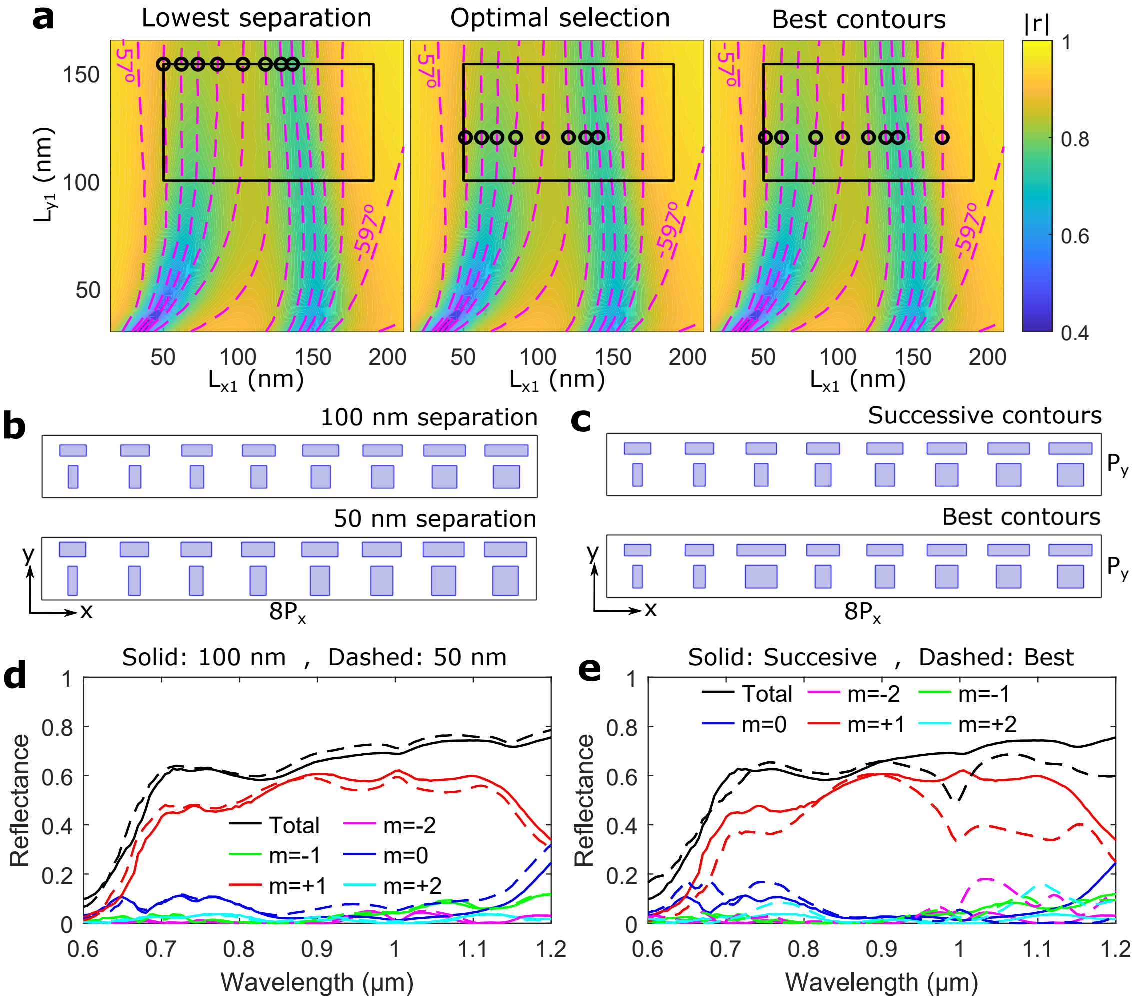

Figure S3: Selection of nanobrick dimensions for supercell modeling. (a) Color maps of calculated reflection coefficient amplitude with imposed contours representing reflected phase for DGSPR unit cells for system parameters of $P_x = P_y = 330\,\text{nm}$, $t_m = t_s = 70\,\text{nm}$, $d_x = 90\,\text{nm}$, $t_b = g = 50\,\text{nm}$, $\lambda_0 = 900\,\text{nm}$ and normal incident TM polarized light. The separation between phase contours is 45°. (b,c) Supercell sketches for beam steering along the positive x-direction for TM polarization. (d,e) Calculated diffraction efficiencies for orders $|m| \leq 2$ as a function of the wavelength of incident light for TM polarization and (d) for investigation of separation to nanobricks in neighbor supercells as well as (e) for selecting nanobrick dimensions on successive phase contours or by largest reflection coefficient amplitude.

suppress unintended diffraction orders around the design wavelength has worsened, leading to the choice of using the 100 nm separation approach (Figure S3d).

The $> 2\pi$ reflected phase range leads to multiple phase contours corresponding to the same phase, thereby introducing a new aspect to consider when selecting nanobricks for supercell modeling. A comparison is made between selecting nanobricks on successive phase contours opposed to simply selecting for better reflection coefficient amplitude. A choice of nanobricks on successive contours leads to each unit cell having an environment that

is closer to the periodic array of identical unit cells used in the simulations that produce the phase-amplitude maps (Figure S3a), whereas larger reflection coefficient amplitude leads to less optical loss. For comparison of both approaches, eight-element supercells are designed by sequentially placing eight elements, indicated by black circles (middle and left panels of Figure S3a), to form supercells (Figure S3c). The available reflected phase range results in three phase contours more than required, opening the possibility to optimize elements 1-3 based on reflection coefficient amplitude, but only the third element showed improved reflection amplitude and is different between the two supercells. Simulations of the designed metasurfaces show that the performance at the design wavelength is similar for both approaches, however the broadband performance is superior for the nanobrick dimensions on successive contours (Figure S3e). Concluding our considerations regarding optimal design rules when selecting nanobrick dimensions, we conclude that the optimal set of design rules are to select nanobrick dimensions on successive phase contours for a separation to nanobricks in neighbor supercells of twice the minimum value.

4 Another approach to avoid duplicating nanobricks

The performance of single-resonator metasurfaces is limited by the requirement to duplicate nanobricks. One approach to avoid this is the heterogeneous metasurface visited in the main text (Figure 6). A second approach is to smoothly cover the available phase range ignoring the missing phase. This results in uneven phase steps in the phase-gradient. The single-resonator unit cell offers close to 290° of available reflected phase range, but it takes 315° to evenly cover the entire 2π phase range with an eight-element supercell. The given reflected phase range allows for eight elements evenly spaced in phase with a separation of 41.4°; slightly less than the ideal 45° (Figure S4a). The selected elements (black circles) are combined in a supercell (Figure S4b), which is modeled for both TM and TE polarization (Figure S4c). Simulations of the metasurface show that the performance is close to identical for both polarizations and displays a reflectance of $> 80\,\%$ close to the design wavelength. The broadband performance is decent with efficiency reducing further from the design wavelength, so it does not show the nice even reflectance as seen for the eight- and 16-element DGSPR metasurfaces. The metasurface smoothly covering the available phase range shows good performance, overcoming the main problem when duplicating elements, namely the increase in unintended diffraction orders. However, the

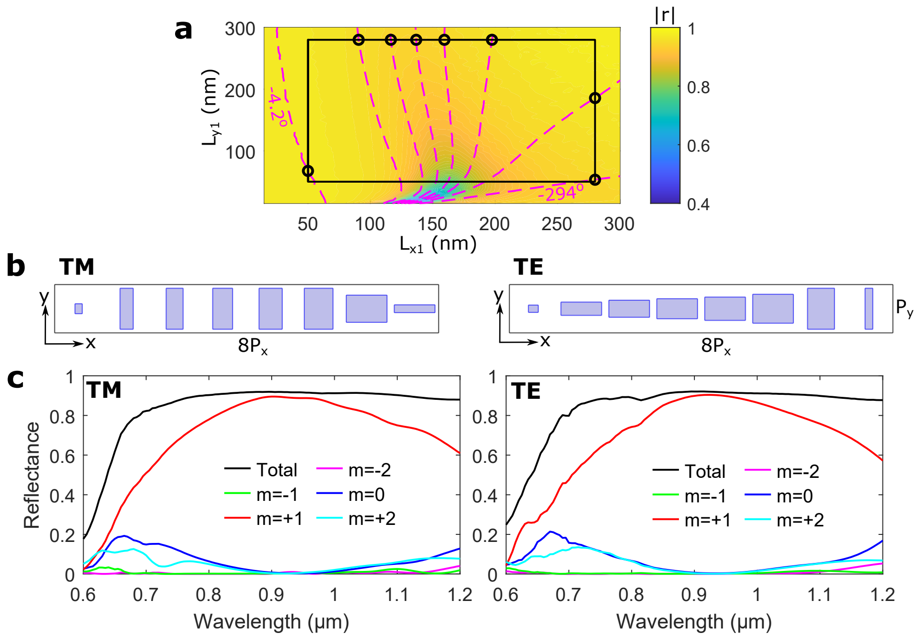

Figure S4: Calculated performance for an eight-element single-resonator supercell smoothly covering the available phase. (a) Color map of calculated reflection coefficient amplitude with imposed contours representing reflected phase for system parameters of $P_x = P_y = 330\,\text{nm}$, $t_m = t_s = 70\,\text{nm}$, $t_b = 50\,\text{nm}$, $\lambda_0 = 900\,\text{nm}$ and normal incident TM polarized light. The separation between phase contours is 41.4°. (b) Supercell sketches for beam steering along the positive x-direction for both TM and TE polarization. (c) Calculated diffraction efficiencies for orders $|m| \leq 2$ as a function of wavelength of incident light for TM and TE polarization.

difference between the applied and the ideal phase step is also only approximately 8 %.

5 Experimental verification of the DGSPR metasurface

As discussed in the main text, two DGSPR metasurfaces designed for operation with TM and TE polarized light at 900 nm were fabricated. Scanning electron microscopy (SEM) images indicate good correspondence between the designed and actual nanobricks (Figure S5a), but the fabricated nanobricks show some discrepancies to the designed nanobricks, e.g. the rounded corners of the ideally box-shaped nanobricks and deviations in dimensions (Figure 7a). Furthermore for TM polarization, some of the larger nanobricks have merged within their respective unit cell.

After fabrication the DGSPR metasurfaces are optically characterized to investigate their performance. An incident low-power continuous wave laser beam is weakly focused on the sample by a long working distance 20X objective. The reflected light is collected by the same objective, split from the incident light using a beam splitter (BS1) and imaged

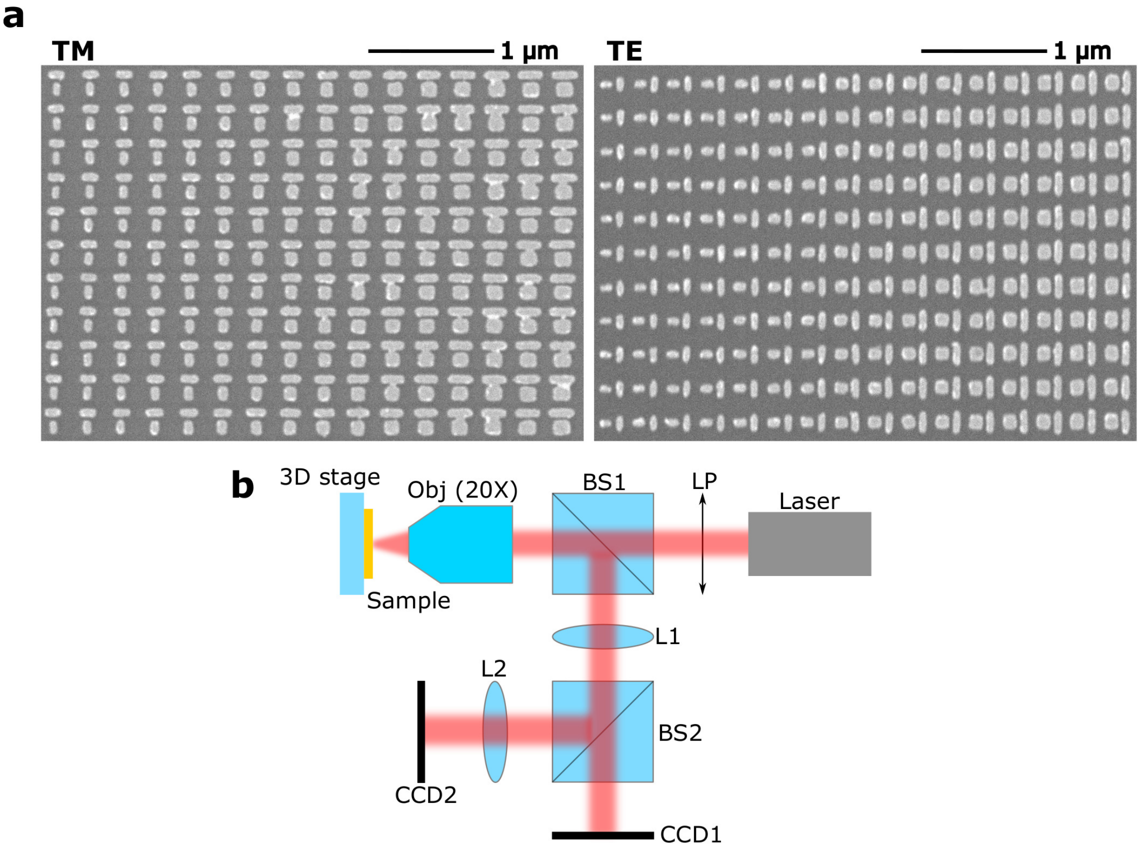

Figure S5: Experimental verification of the DGSPR metasurface. (a) SEM images of the fabricated DGSPR metasurfaces for TE and TM polarization. Each image displays an area of 1×11 supercells, designed to diffract the incoming light into the $+1^{\text{st}}$ diffraction order. (b) Schematic of the optical setup used for characterization of the DGSPR metasurfaces. LP: Linear polarizer, BS: Beam splitter, Obj: Objective, L: Lens, CCD: Charged coupled device.

in the real and Fourier planes on charged coupled devices (CCD1 and CCD2, respectively) (Figure S5b).

References

[1] M. G. Nielsen, D. K. Gramotnev, A. Pors, O. Albrektsen, and S. I. Bozhevolnyi, "Continuous layer gap plasmon resonators," *Opt. Express*, vol. 19, pp. 19310–19322, Sep 2011.
